# Mitral Annulus Dynamics: Another Unmet Need in Valvular Heart Disease

**DOI:** 10.1111/echo.70352

**Published:** 2025-11-20

**Authors:** Paula Cristina Morariu, Mariana Floria, Diana‐Elena Floria, Daniela Maria Tanase

**Affiliations:** ^1^ Faculty of Medicine Department of Internal Medicine University of Medicine and Pharmacy "Grigore T. Popa" Iasi Romania; ^2^ Emergency Clinical Hospital "Saint Spiridon" Iasi Romania

1

Three‐dimensional (3D) transthoracic and transesophageal echocardiography (TEE) are essential tools that provide detailed anatomical and functional insights, fundamental for both diagnosis and therapeutic planning in the assessment of structural disease [1]. In addition, the possibility to visualize 3D valve live images with high spatial and temporal resolution is essential in intraoperative and interventional settings. These imaging modalities enable the acquisition of the most complete dataset to describe the entire valvular apparatus and to guide structural heart interventions [2]. A major challenge in transcatheter and structural heart procedures planning is the limited ability to predict the anatomic and hemodynamic effects of a device prior to its placement. Three‐dimensional echocardiography allows for comprehensive evaluation of changes in mitral annular size, shape, and motion, defined as mitral annulus dynamics.

The recently published manuscript of Shaaban et al. [3] tries to bring new data about postoperative hemodynamic changes in mitral annulus dynamics in patients with hypertrophic cardiomyopathy. This clinical study evaluated the mitral valve annulus's dynamics and the effect of septal myectomy on its geometry and function using 3D TEE in patients with hypertrophic obstructive cardiomyopathy. Before and after surgery, the following mitral annular parameters were compared in the study and control groups: annulus dimensions (anteroposterior, anterolateral‐posteromedial, intertrigonal and intercommissural diameter, perimeters, height, sphericity index), angles (aorto‐mitral, non‐planar), 3D area and saddling (the degree of saddle‐shaped deformation of the mitral annulus). They concluded that, preoperatively, in these patients, the mitral valve annulus exhibits reduced dynamic motion, with a notable loss of normal systolic antero‐posterior contraction and saddling [3]. Postoperatively, no significant changes were detected in mitral annulus dynamics throughout the four phases of the cardiac cycle. Patients with hypertrophic obstructive cardiomyopathy demonstrated significantly larger mitral annular dimensions compared with controls, particularly in the antero‐posterior and anterolateral–posteromedial diameters, as well as in the 3D annular area and perimeter [3]. Additionally, they exhibited a less obtuse aorto‐mitral angle, which may contribute to the development of left ventricular outflow tract obstruction through altered valvular mechanics. Furthermore, hypertrophic obstructive cardiomyopathy patients showed reduced dynamic motion of the mitral annulus, characterized by decreased systolic anteroposterior contraction and a diminished saddle‐shaped configuration [3]. Immediate postoperative echocardiographic evaluation revealed effective relief of left ventricular outflow tract obstruction. No concurrent acute improvement in mitral annular dynamics was observed, underscoring the complex relationship between surgical intervention and mitral valve geometry [3].

The primary structural deformities of the mitral apparatus could be the primordial causes of systolic anterior motion in patients with hypertrophic cardiomyopathy. These primary abnormalities include leaflet elongation (including increased size of both anterior and posterior leaflets or asymmetrical enlargement of either the anterior leaflet or a posterior leaflet scallop), papillary muscle displacement, abnormal coaptation, and chordal slack [4]. However, it is not well known how these deformities cause abnormal hemodynamic changes during systole in the left ventricular outflow tract. Since the morphology and position of structural deformities can change dynamically during systole and diastole, these shifts in the mitral apparatus during systole may significantly contribute to the formation of a hemodynamic obstruction in the left ventricular outflow tract [4]. These patients have shorter coaptation‐septal distance, shorter inter‐papillary muscles distance, and larger mitral tenting volume/body surface area. In addition, the left ventricular outflow tract pressure gradient, in these patients, increases with mitral tenting volume/body surface area and decreases with coaptation‐septal distance and the inter‐papillary muscles distance at mid‐systole but not at mid‐diastole [4]. The coaptation‐septal distance is closely associated with the left ventricular outflow tract pressure gradient. Therefore, it seems that dynamic geometric changes by interaction of papillary muscles, mitral tenting, and the coaptation point at mid‐systole are important contributors to left ventricular outflow obstruction in patients with hypertrophic cardiomyopathy presenting with systolic anterior motion [4]. It is important to study mitral annulus dynamics preoperatively and postoperatively. A combination of an appropriate septal myectomy and, if necessary, the correction of the mitral apparatus may lead to the best results with a low operative risk [4].

The mitral annular dynamics can be evaluated across the cardiac cycle using dedicated software and Doppler‐derived pressure gradients. After manually placing landmarks on the anterior and posterior points of the mitral annulus and the coaptation point, the software then semi‐automatically tracks mitral annulus motion throughout the cardiac cycle, generating a dynamic 3D model of the mitral valve [3]. The assessment of mitral valve shape on transmitral hemodynamics was studied in patients with valvular heart disease and hypertrophic cardiomyopathy, in relation to left ventricular and left atrial function [5]. The mitral annulus dynamics have been studied, including on reduced‐order models [6]. Immediate and long‐term follow‐up data from septal reductive interventions, performed without corrective manipulation of the mitral apparatus, showed favorable outcomes in both hemodynamics and subjective symptoms [4]. However, this topic is still discussed as incompletely studied in relation to the results of interventional procedures.

Three‐dimensional transesophageal echocardiography serves as a valuable tool for guiding surgeons in assessing septal geometry, thereby facilitating the precise identification of the site, extent, and depth required for myectomy during surgery. Moreover, 3D echocardiography enables quantification of the resected septal mass, optimizing surgical outcomes, and provides essential post‐resection evaluation of left ventricular outflow tract patency. Three‐dimensional echocardiography, transthoracic, and TEE have become essential to identify intrinsic mitral valve pathology frequently observed in hypertrophic cardiomyopathy. His ability to assess the geometric dynamics of the mitral apparatus is permitted by a complete and direct visualization of abnormalities of the mitral valve apparatus, including hypertrophy of the papillary muscles and their anterior displacement, and intrinsic increase in mitral leaflet area and elongation [7]. The displacement of the mitral coaptation point toward the septum, reduced interpapillary muscle distance during mid‐systole, and increased mitral tenting were interrelated factors associated with left ventricular outflow tract obstruction and systolic anterior motion.

Nowadays, predicting the valvular hemodynamic outcomes in intraoperative and interventional heart procedures is challenging. The mitral annulus is not a static, simple ring, but a complex, non‐planar, and dynamic structure that changes shape throughout the cardiac cycle. Its movement is tightly coupled with left ventricular function. Traditional diagnostic and therapeutic approaches often focus on static measurements, failing to fully account for the dynamic changes in mitral annulus size, shape, and motion. There is a significant need for more accurate imaging to assess mitral annulus dynamics throughout the cardiac cycle and for a more comprehensive understanding of how these dynamics influence disease and impact treatment outcomes. This includes developing new technologies and treatment strategies, possibly with a hybrid approach, to better manage this complex patient population. More research, like multiphase assessment of mitral annular dynamics, is needed to understand the specific pathophysiology of the annulus in valvopathies to pave the way for better treatments [8]. The recently published study of Shaaban et al. [3] brings to the fore, once again, the mitral annulus dynamics, another possible unmet need in valvular heart disease.

## Conflicts of Interest

The authors declare no conflicts of interest.

## Data Availability

The authors have nothing to report.
